# Comparative Study on Blood Gas Indicators, Antioxidant Capacity, Intestinal Metabolome, and Microbiome in High- and Low-Performance Tumbler Pigeons

**DOI:** 10.3390/biology15141193

**Published:** 2026-07-20

**Authors:** Xiangxi Liu, Xiaoyu Zhao, Haiying Li, Yingping Wu, Yingying Yao, Zening Wang

**Affiliations:** College of Animal Science, Xinjiang Agricultural University, Urumqi 830000, China; 17799623750@163.com (X.L.); lhy-3@163.com (H.L.); wyp_941208@163.com (Y.W.); yyy1234560317@163.com (Y.Y.); 15730942854@163.com (Z.W.)

**Keywords:** tumbler pigeons, blood gas indicators, antioxidant indicators, intestinal metabolites, intestinal microbiota

## Abstract

The purpose of this experiment is to investigate the differences in blood gas indicators, antioxidant indicators, and lactate content between high- and low-performance tumbler pigeons after exercise, and to use metabolomics and metagenomics techniques to screen for differential metabolites and bacteria related to tumbler pigeon exercise. This experiment selected 12 high-performing (HP) and 12 low-performing (LP) tumbler pigeons, half male and half female, and all pigeons were raised under the same conditions. Three experimental pigeons were grouped for exercise training, with a 20 min training session. The results showed significant differences (*p* < 0.05) in multiple blood gas parameters related to acid–base balance and gas exchange between the HP and LP groups after tumbler pigeon exercise. In addition, the content of glutathione peroxidase (GSH Px), superoxide dismutase (SOD), and catalase (CAT) in the liver of the HP group of tumbler pigeons was significantly lower than that of the LP group (*p* < 0.05). In comparison, the content of malondialdehyde (MDA) was significantly higher than that of the LP group (*p* < 0.05). The content of lactate (Lac) was significantly higher than that of the LP group (*p* < 0.05). Non-targeted metabolomics analysis revealed that differential metabolites were mainly enriched in pathways such as biosynthesis of unsaturated fatty acids, starch and sucrose metabolism, and fatty acid synthesis. Intestinal metagenomics analysis revealed that the *Corynebacteriaceae*, *Bacillus*, *Pseudomonad phylum* and *Corynebacterium* were significant biomarkers with significant differences in the gut microbiota of the HP group of tumbler pigeons (*p* < 0.05). In summary, there are significant differences in blood physiological parameters, antioxidant capacity, lactate content, intestinal metabolites, and gut microbiota between high- and low-performance tumbler pigeons after exercise. This result provides theoretical guidance and data support for cultivating high-performance tumbler pigeons.

## Simple Summary

Under competitive conditions, tumbler pigeons show significant individual differences in flight capacity and recovery ability following strenuous exercise, yet the physiological basis of these disparities remains unclear. This study compared blood gas indicators, antioxidant biomarkers, intestinal metabolites, and gut microbiota between high-performance and low-performance tumbler pigeons after exercise. The results revealed differences in blood physiological status, oxidative status, intestinal metabolites, and gut microbial communities between the two groups. Notably, high-performance tumbler pigeons exhibited lower antioxidant enzyme activity and higher malondialdehyde (MDA) levels. This study provides theoretical basis for explaining differences in flight performance and contributes to the selective breeding of high-performance tumbler pigeons.

## 1. Introduction

In recent years, China’s pigeon racing sport has experienced robust growth, accompanied by a steady annual increase in the number of pigeon breeders [1]. Tumbler pigeons are renowned for their ability to navigate long distances back to their base, while soaring pigeons are famous for their continuous aerial somersaults [2]. The tumbler Pigeon is a local specialty breed characterized by distinctive aerial behaviors, such as circling in the air and performing continuous flips [3]. The short-term, high-intensity exercise of tumbler pigeons places significant demands on the body’s oxidative stress levels, energy metabolism, and intestinal microbial flora stability. However, based on the current competition results, there are significant differences in the movement performance and post-exercise recovery ability of the tumbler pigeons. During the flight of tumbler pigeons, a large amount of energy is consumed to sustain the flight activity. The energy mainly comes from the metabolism of sugar and fat.

However, during high-intensity exercise, the body experiences a condition in which oxygen consumption exceeds intake, leading to increased glycolysis. This process involves the regulation of numerous metabolic enzymes to ensure the stable supply of energy during the flight [4,5]. Peng et al. [3] found that during the flight of the tumbler pigeon, the fatty acid metabolism pathway was upregulated, gluconeogenesis was downregulated, and the stress response was enhanced. Previous studies have shown that compared with the non-athlete control group, elite rugby players have a higher microbial diversity and short-chain fatty acid levels in their gut microbiota [6,7]. Athletes at higher competitive levels often have greater abundances of microorganisms and a greater potential for carbohydrate metabolism [8,9]. In addition to metabolic levels and gut bacteria, the researchers also found that blood gas indicators and antioxidant levels are closely related to the exercise process. He et al. [10] conducted a study indicating that monitoring changes in blood gas indicators and electrolyte levels could provide a scientific basis for promoting the body’s recovery. Li [11] found in his study that after Mongolian horses engaged in 20 km of high-intensity exercise, the content of malondialdehyde in their blood significantly increased, while the total antioxidant capacity, superoxide dismutase, and glutathione peroxidase contents significantly decreased. The above research indicates that the differences in an animal’s athletic performance are often accompanied by changes in its antioxidant levels, metabolism, and gut microbiota. Currently, there are few reports on the correlation between physiological metabolism and the gut microbiota of pigeons after exercise.

Therefore, this study aims to investigate physiological differences between high-performance and low-performance tumbler pigeons after intense exercise. By comparing blood gas, antioxidant biomarkers, intestinal metabolomics and gut metagenomics data, we seek to (1) explore post-exercise changes in oxidative stress and blood gas status; (2) identify differential metabolites and gut microbial communities associated with flight performance; (3) screen for potential multi-omics pathways linked to divergent flight capacity in tumbler pigeons. These findings will provide fundamental data and theoretical references for the breeding of high-performance tumbler pigeons.

## 2. Materials and Methods

### 2.1. Animals and Experimental Design

In this experiment, 150 pigeons aged 2–3 years with a body weight of 250 ± 30 g were selected for the measurement of their physical performance. Based on the test results, the pigeons were divided into two groups: a high movement performance (HP) group and a low movement performance (LP) group. Those pigeons with more than 5 flips and each flip having more than 4 rotations were classified as high-performance tumbler pigeons. The test results showed that 80 tumbler pigeons were classified into the high-performance group, while 70 were assigned to the low-performance group. During the training period, all the pigeons were maintained under the same feeding and management conditions. Before the formal training began, 12 samples of tumbler pigeons were randomly selected from each group, and pigeon rings were attached to their feet. These pigeons were used to test blood gas indicators, antioxidant capacity, intestinal metabolic profiles, and metagenomics. The tumbler pigeons were trained in groups of three. Each training session lasted for 20 min. During the training, two professionals recorded, in real time, the number of flips and the total number of flip cycles for the experimental pigeons. The pigeons used in the experiment and the training grounds were provided by the Lifeng Breeding Cooperative in Yanqi County, Baizhong City, Xinjiang.

### 2.2. Sample Collection and Processing

After the selected experimental pigeons reached a state of fatigue following the flight training, while the pigeons were still alive, 2 milliliters of blood samples were collected from the lower veins of the wings through a one-time vacuum tube. The collected whole blood was centrifuged at 4000 rpm for 5 min, and the supernatant serum was retained for subsequent tests. After the blood collection, the pigeons were humanely euthanized by cervical dislocation, and the liver tissue on the same side was immediately collected and then stored at −80 °C for further analysis.

### 2.3. Blood Gas Parameters

According to the manufacturer’s instructions, blood gas and electrolyte parameters were measured using a veterinary blood gas analyzer (VG2, Seamaty Technology Co., Ltd., Chengdu, China) equipped with a disposable dry electrochemical reagent kit. After preparing the serum, immediately place the sample into the test kit and conduct the analysis immediately to minimize the changes caused by storage. The measured blood gas parameters include potential of hydrogen (pH), partial pressure of venous carbon dioxide (PvCO_2_), partial pressure of oxygen (PvO_2_), sodium ion (Na^+^), potassium ion (K^+^), chloride ion (Cl^−^), calcium ion (Ca^2+^), hematocrit (Hct), total carbon dioxide (TCO_2_), hemoglobin (Hb), bicarbonate (HCO_3_^−^), base excess (BE), and oxygen saturation (SvO_2_), etc. The average time required for each pigeon to have its blood drawn and analyzed is approximately 2–3 min. All the test results will be automatically uploaded to the computer for subsequent statistical analysis.

### 2.4. Antioxidant Properties

After the pigeons’ physical strength was exhausted during the experiment, they were euthanized by cervical dislocation. Immediately after death, the liver on the same side was collected and separately stored at −80 °C for preservation. The total antioxidant capacity, glutathione peroxidase activity, superoxide dismutase activity, and malondialdehyde content were determined according to the instructions of the corresponding reagent kits. The reagent kits were purchased from Sang gon Biotech Co., Ltd. (Shanghai, China)Remove the required strips from the aluminum foil bag that has been balanced at room temperature for 20 min. The remaining strips should be sealed in a self-sealing bag and returned to 4 °C. Set up the standard sample wells and the sample wells. Add 50 μL of standard solutions at different concentrations to each standard sample well. First, add 10 μL of the sample to be tested into the sample wells, then add 40 μL of the sample diluent. The blank wells do not receive any addition. Except for the blank wells, 100 μL of horseradish peroxidase (HRP)-labeled detection antibody was added to each standard and sample well. The reaction wells were sealed with a sealing film and incubated at 37 °C in a water bath or an incubator for 60 min. Remove the liquid and blot dry with absorbent paper. Fill each well with the washing solution, let it stand for 1 min, then remove the washing solution and blot dry with absorbent paper. Repeat this process 5 times (or use a plate washer for washing the plate). Add 50 μL of substrate A and 50 μL of substrate B to each well, and incubate at 37 °C in the dark for 15 min. Add 50 μL of the stop solution to each well, and measure the OD at 450 nm within 15 min. In the Excel worksheet, use the standard sample concentration as the abscissa and the corresponding OD value as the ordinate to plot the linear regression curve for the standard sample. Then, calculate the concentration of each sample using the curve equation.

### 2.5. Metabolomics Analysis

Tissue samples ground with liquid nitrogen (100 mg) were transferred to an EP tube, followed by the addition of 500 μL of 80% aqueous methanol. The mixture was vortexed thoroughly and incubated on ice for 5 min. After centrifugation at 15,000× *g* for 20 min at 4 °C, an aliquot of the supernatant was collected and diluted with ultrapure water to a final methanol concentration of 53%. The diluted solution was centrifuged again under identical conditions, and the resulting supernatant was retained for subsequent LC-MS/MS detection. LC-MS/MS detection was performed by Novogene Biotechnology Co., Ltd. (Beijing, China). Chromatographic separation was carried out on a Hypersil Gold C18™ column. The mobile phase consisted of 0.1% formic acid in water (mobile phase A) and pure methanol (mobile phase B). Electrospray ionization (ESI) was applied for mass spectrometry detection in both positive and negative ion modes.

### 2.6. Metagenomics Analysis

Genomic DNA (1 μg) was fragmented via sonication to yield 350 bp inserts. Subsequent end repair, A-tailing, adapter ligation, purification and PCR amplification were conducted to construct the sequencing library. Insert fragment sizes were characterized via the AATI system, and library quantification was completed via qPCR (effective concentration > 1.5 nM). Libraries that passed quality control were pooled based on their concentrations and expected data output, followed by PE150 paired-end sequencing on the NovaSeq platform. Raw sequencing reads were quality-filtered with fastp to generate clean data; reads containing adapters, low-quality segments or excess ambiguous N bases were discarded. Host-derived contaminant reads were eliminated via alignment against the tumbler pigeon host reference genome using Bowtie2. De novo assembly of clean reads into scaffolds was accomplished using MEGAHIT, and scaffold segmentation was performed in the downstream workflow. Open reading frame (ORF) prediction was carried out on all scaffolds ≥ 500 bp in length with MetaGeneMark 2.1.

### 2.7. Data Analysis

The identified metabolites were annotated using the KEGG, HMDB, and LIPIDMAPS databases. The metabolomics data processing software metaX 1.4.16 was used to conduct principal component analysis (PCA) and partial least squares discriminant analysis (PLS-DA) on the transformed data. The default criterion for screening differential metabolites is that VIP (Variable Importance in Projection) should be greater than 1, the *p*-value should be less than 0.05, and the fold change (FC) should be greater than or equal to 2 or less than or equal to 0.5. Use the R software ggplot2 (version 3.3.4) to create volcano plots and bubble charts, and use the R software Pheatmap to draw cluster heatmaps. Using the KEGG database to study the functions of metabolites and metabolic pathways, a *p*-value less than 0.05 indicates that the pathway is significantly enriched. After removing fragments shorter than 100 nt, the initial gene catalog was obtained using CD-HIT to remove redundancy. Using Bowtie2 to compare against the clean data, genes with fewer than 2 reads per sample were filtered out, and a gene catalog suitable for subsequent analysis was obtained. Based on DIAMOND, unigenes were compared against NR (bacteria/fungi/archaea/viruses), and results with e ≤ 10^−5^ were selected. Since each sequence may have multiple comparison results, species annotation was completed using LCA. PCA and ANOSIM tests using R were used to examine the differences in communities between groups; LEfSe (with LDA > 3) was used to screen for differentially expressed species; The unigenes were compared with the KEGG, eggNOG, and CAZy databases using DIAMOND, and the complete functional annotation with the highest score was selected. The sequencing and data analysis were assisted by Novogene Biotechnology (Beijing, China).

The original experimental data were sorted and summarized in Excel; an independent two-sample *t*-test was conducted in SPSS 27.0. Before the test, the normal distribution of all detection indicators was verified. The comparison groups were high-performance tumbler pigeons (HPs) and low-performance tumbler pigeons (LPs). The comparison indicators included blood gas, antioxidant, lactate, metabolic group, and macrogenome-related detection parameters. This study reported significant differences when *p* < 0.05; in the charts, the precise *p* values were presented as much as possible, and only very small *p* values were uniformly labeled as *p* < 0.001. All quantitative detection results were presented as the mean ± standard error of the mean (SEM). We further performed Benjamini–Hochberg false discovery rate (FDR) multiple-testing correction in R to control for type I error caused by mass spectrometry. Metabolites with adjusted FDR < 0.05 and fold change > 1.2 or <0.833 were defined as significantly differentially expressed metabolites. For single physiological and microbial alpha-diversity indices, intergroup differences were identified at raw *p* < 0.05 without multiple correction. The experimental charts were generated using GraphPad Prism 8.0.2; the correlation analysis of metabolites was performed using the Spearman correlation coefficient.

## 3. Results

### 3.1. Comparative Analysis of High and Low Performance Tumbler Pigeons’ Blood Gas Indicators

As shown in Table 1, the PO_2_ level in the bodies of the tumbler pigeons in the HP group was significantly lower than that in the LP group (*p* < 0.05); while the levels of Hct and Hb concentration in the bodies of the tumbler pigeons in the HP group were extremely significantly higher than those in the LP group (*p* < 0.05); there were no significant differences in the other indicators between the HP and LP groups (*p* > 0.05).

### 3.2. Comparative Analysis of Antioxidant Indexes in the Liver of High and Low Performance Tumbler Pigeons

As shown in Table 2, the contents of GSH-Px, SOD, and CAT in the livers of the pigeons in the HP group were significantly lower than those in the LP group (*p* < 0.05), while the MDA and lactate contents were significantly higher than those in the LP group (*p* < 0.05). There was no significant difference in T-AOC and TP contents between the two groups (*p* > 0.05).

### 3.3. Differences in Gut Metabolites After High and Low Physical Activity in Tumber Pigeons

**Figure 1:**
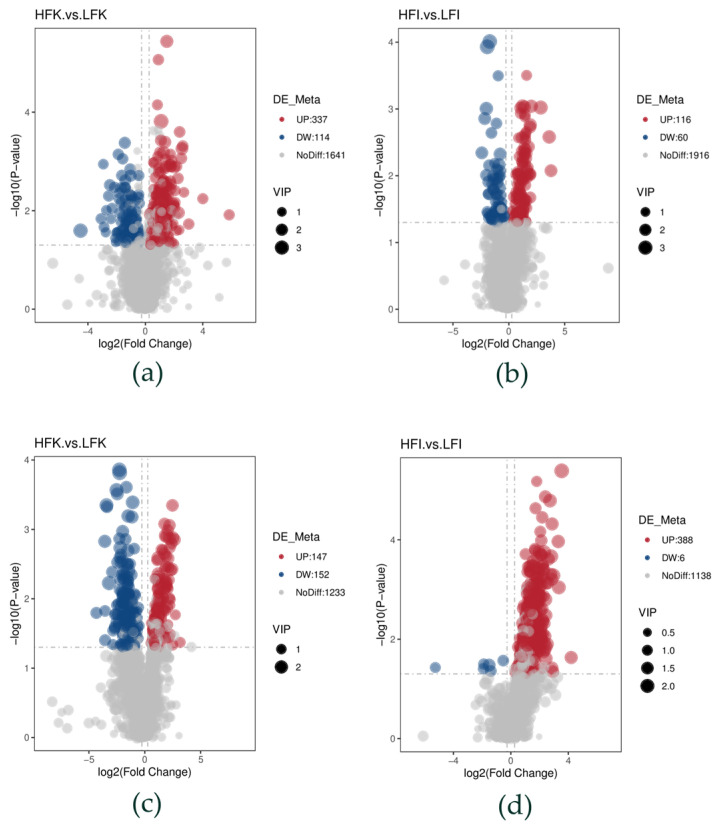
Volcano plot in positive and negative ion mode. HFK: HP group tumbler pigeon jejunum, HFI: HP group tumbler pigeon ileum, LFK: LP group tumbler pigeon jejunum, LFI: LP group tumbler pigeon ileum, the same below. (**a**) Volcano plot of differential metabolites between HP group tumbler pigeon jejunum and LP group tumbler pigeon jejunum in positive ion mode; (**b**) Volcano plot of differential metabolites between HP group tumbler pigeon ileum and LP group tumbler pigeon ileum in positive ion mode; (**c**) Volcano plot of differential metabolites between HP group tumbler pigeon jejunum and LP group tumbler pigeon jejunum in negative ion mode; (**d**) Volcano plot of differential metabolites between HP group tumbler pigeon ileum and LP group tumbler pigeon ileum in negative ion mode. Red circles: significantly upregulated metabolites; blue circles: significantly downregulated metabolites; gray circles: metabolites without significant difference. The size of each circle represents the VIP value. The horizontal dashed line corresponds to *p* = 0.05, and the two vertical dashed lines represent fold change thresholds of 1.2 and 0.833.

**Figure 2:**
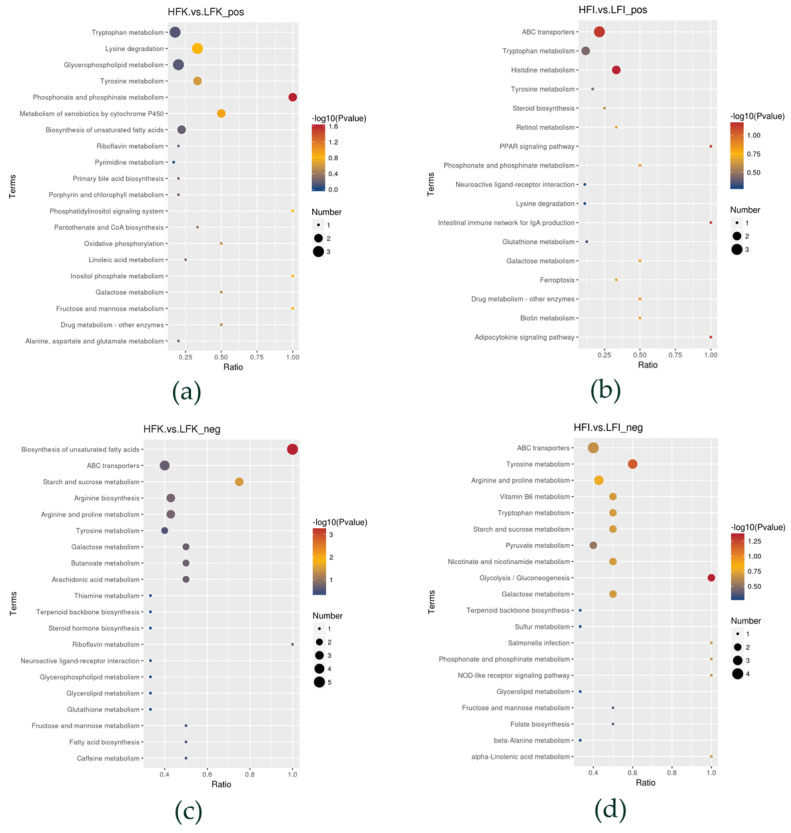
Bubble chart of KEGG enrichment under positive and negative ion modes. (**a**) Enrichment bubble plot of metabolic pathways between HP group tumbler pigeon jejunum and LP group tumbler pigeon jejunum in positive ion mode; (**b**) Enrichment bubble plot of metabolic pathways between HP group tumbler pigeon ileum and LP group tumbler pigeon ileum in positive ion mode; (**c**) Enrichment bubble plot of metabolic pathways between HP group tumbler pigeon jejunum and LP group tumbler pigeon jejunum in negative ion mode; (**d**) Enrichment bubble plot of metabolic pathways between HP group tumbler pigeon ileum and LP group tumbler pigeon ileum in negative ion mode. The horizontal axis represents the enrichment ratio of each pathway. The vertical axis displays the annotated metabolic pathway terms. The color gradient of dots corresponds to −log10 (*p* value), and the dot size stands for the number of enriched differential metabolites contained in each pathway.

In this study, PCA was employed to process the detection data. The results showed that the sample points clustered well, indicating that the experiment’s quality control was reliable. Meanwhile, the samples in the HP and LP groups showed a distinct separation trend, suggesting significant differences in the pigeons’ intestinal metabolic profiles based on exercise performance (Figure S1). Further, using Partial Least Squares Discriminant Analysis (PLS-DA), it was demonstrated that the model has high explanatory power and excellent predictive performance. The model’s reliability was evaluated using permutation tests, which confirmed there was no overfitting. These results indicated significant differences in the metabolic profiles of the posterior jejunum and ileum in tumbler pigeons with different exercise performance levels (Figure S2). The screening criteria were set to metabolites with VIP (Variable Importance in Projection) > 1.0, FC > 1.2 or FC < 0.833, and a *p*-value < 0.05 as significant. In the positive ion mode, 451 different metabolites were finally identified in the cecal contents of the HP group and the LP group, among which 337 were upregulated and 114 were downregulated (Figure 1a); 176 different metabolites were finally identified in the ileum, of which 116 were upregulated and 60 were downregulated (Figure 1b). In the negative ion mode, 299 different metabolites were finally identified in the cecal contents of the HP group and the LP group, among which 147 were upregulated and 152 were downregulated (Figure 1c); 394 different metabolites were finally identified in the ileum, of which 388 were upregulated and 6 were downregulated (Figure 1d). Some of the differential metabolites are shown in Table S1. Cluster analysis was performed on the selected differential metabolites. The results of the cluster heatmap showed a trend of change across all groups. The differential metabolites in the intestines of each group showed clear groupings, indicating significant differences in the types and levels of metabolites among the groups (Figure S3). To investigate the metabolic pathways underlying differential metabolites and their correlations, a metabolic pathway enrichment analysis was conducted for metabolites with significant differences. Based on the enrichment analysis results, the 20 metabolic pathways with the smallest *p*-values were selected to create a bubble chart (Figure 2). The results showed that in the positive ion mode, the differential metabolites in the jejunum of the HP group and LP group were mainly enriched in the pathways of phosphate and subphosphate metabolism (*p* < 0.05) (Figure 2a); in the negative ion mode, the differential metabolites in the jejunum of the HP group and LP group were mainly enriched in the pathways of unsaturated fatty acid biosynthesis, starch and sucrose metabolism, etc. (*p* < 0.05) (Figure 2c). In contrast, the differential metabolites in the ileum were mainly enriched in the pathways of glycolysis, etc. (*p* < 0.05) (Figure 2d).

### 3.4. Differences in Gut Microbial Communities Between High and Low Physical Performance Tumbler Pigeons

**Figure 3:**
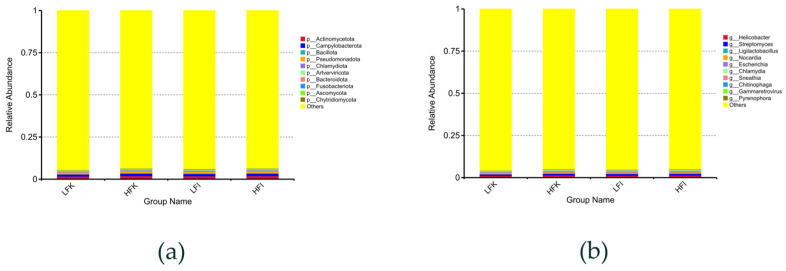
Bar chart of relative abundance at the family and genus levels. (**a**) Stacked bar chart of species relative abundance at family level; (**b**) Stacked bar chart of species relative abundance at genus level. The vertical axis represents relative abundance of microbiota, and the horizontal axis shows sample groups. Different colored segments correspond to distinct microbial taxa, and the yellow segment Others aggregates all taxa with low relative abundance.

**Figure 4:**
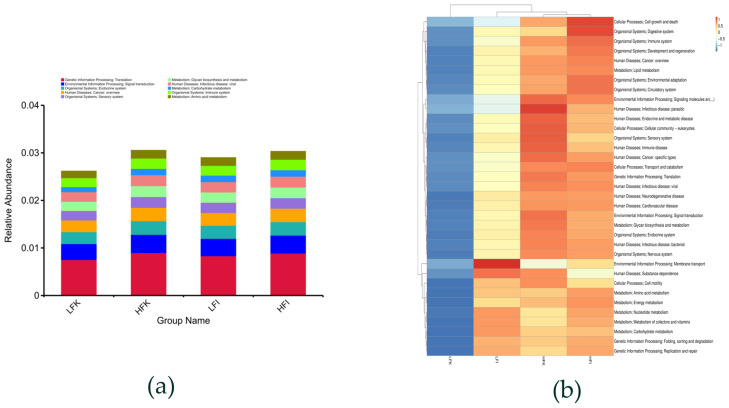
(**a**) KEGG database annotation diagram; (**b**) KEGG enrichment analysis clustering heatmap. The vertical axis of panel (**a**) represents the relative abundance of functional pathways, and the horizontal axis shows groups. Different-colored segments represent distinct KEGG functional categories. The color gradient in panel (**b**) reflects the enrichment level of each KEGG pathway.

**Figure 5:**
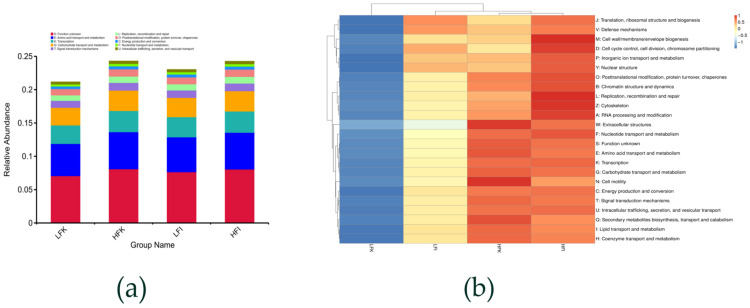
(**a**) Annotation diagram of the eggNOG database; (**b**) clustering heatmap of eggNOG enrichment analysis. The vertical axis of panel (**a**) represents the relative abundance of eggNOG functional entries, and the horizontal axis shows groups. Different−colored segments represent distinct eggNOG functional categories. The color gradient in panel (**b**) reflects the enrichment level of each EggNOG functional entry.

**Figure 6:**
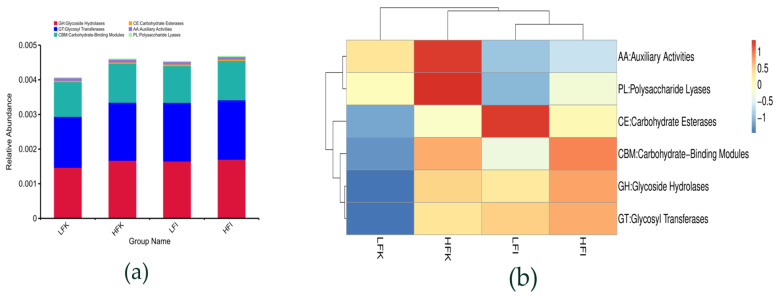
(**a**) Annotation diagram of the CAZy database; (**b**) clustering heatmap of CAZy enrichment analysis. The vertical axis of panel (**a**) represents the relative abundance of CAZy functional modules, and the horizontal axis shows groups. Different−colored segments represent distinct CAZy enzyme categories. The color gradient in panel (**b**) reflects the enrichment level of each CAZy functional module.

**Figure 7:**
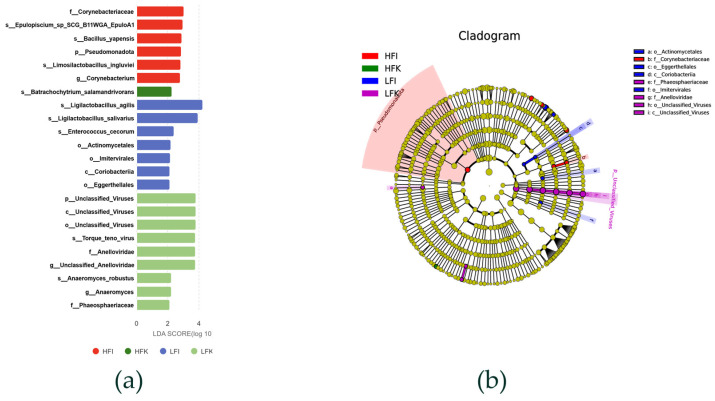
(**a**) LEfSe analysis of intestinal microbiota in HP and LP tumbler pigeon groups; (**b**) phylogenetic tree. The horizontal axis of Figure (**a**) represents the LDA score of microbial groups, while the vertical axis represents the annotated names of the microorganisms. Different-colored bars represent the biomarker groups enriched in the corresponding groups. In Figure (**b**), the color gradient reflects the grouping origin of each microbial group on the evolutionary tree.

**Figure 8:**
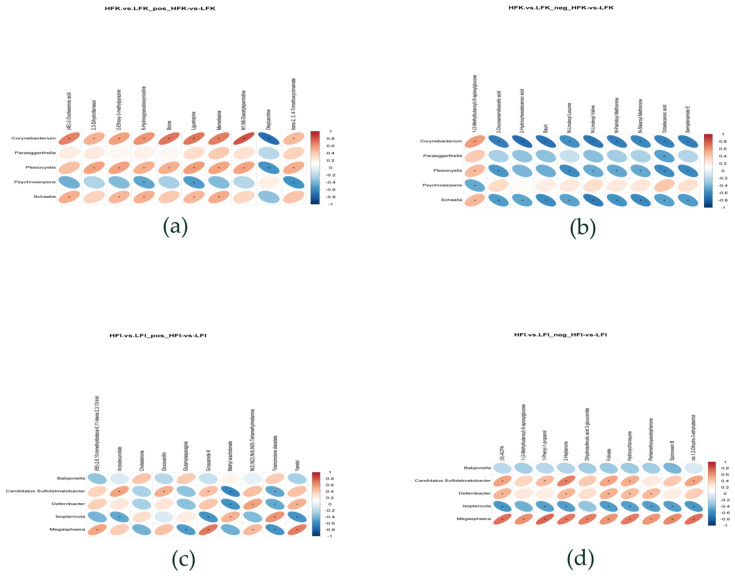
Correlation Heatmap. (**a**) Correlation heatmap of differential metabolites and dominant microbes for jejunum comparison in positive ion mode; (**b**) Correlation heatmap of differential metabolites and dominant microbes for jejunum comparison in negative ion mode; (**c**) Correlation heatmap of differential metabolites and dominant microbes for ileum comparison in positive ion mode; (**d**) Correlation heatmap of differential metabolites and dominant microbes for ileum comparison in negative ion mode. The vertical axis of each panel shows the dominant intestinal microbial genera, and the horizontal axis shows the screened differential metabolites. The color gradient of each ellipse reflects the correlation coefficient between microbes and metabolites. Red indicates positive correlation, and blue indicates negative correlation. The asterisk (*) represents a statistically significant correlation between microorganisms and metabolites (*p* < 0.05).

Metagenomic sequencing of the intestinal microbiota of tumbler pigeons with high and low movement performance was conducted, yielding high-quality data. After strict quality control, the clean data for each sample ranged from 6.03 G to 18.71 G. The Q20 and Q30 values remained above 97% and 93%, respectively, indicating that the sequence identification results for the samples were reliable and could proceed to the next analysis (Table S2). The Chao1 index in the jejunum of the HP group was significantly higher than that in the LP group (*p* < 0.05; Table 3). Applying PCA to reduce the dimension of the multivariate dataset (Figure S4), PC1 and PC2 account for 11.21% and 6.67% of the variance in the samples, respectively. In the coordinate space, the samples from the HFK and LFK groups showed both separation and overlap, indicating that the microbial community structures of the two groups also exhibited significant differences in the degree of similarity. According to the results of ANOSIM analysis, there was a significant difference in the intestinal flora structure between the high and low performance groups (R = 0.223, *p* = 0.026, Figure S5), indicating that the intestinal flora composition of individuals with different performance levels showed significant differentiation, and the grouping effect was good. In contrast, the difference in the intestinal flora structure between the high and low performance groups of the tumbler pigeons was not significant (R = −0.015, *p* = 0.451, Figure S5), suggesting that the intestinal flora composition of the high and low performance groups was relatively similar. No obvious differentiation in performance was observed. At the species level, the top 3 dominant phyla in terms of microbial abundance in the cecum and ileum of the high and low performance tumbler pigeons were all *Actinobacteria*, *Campylobacteria*, and *Pseudomonadales* (Figure 3a); at the genus level, the top 3 dominant genera in terms of microbial abundance in the cecum and ileum of the high and low performance tumbler pigeons were *Helicobacter genus*, *Streptomyces genus*, and *Nocardia genus* (Figure 3b). The KEGG database annotation analysis results showed that the significant genes were annotated to the following signaling pathways: translation, signal transduction, endocrine system, cancer, sensory system, glycan biosynthesis and metabolism, infectious diseases, carbohydrate metabolism, immune system, and amino acid metabolism (Figure 4a). The pathways significantly enriched in the small intestine of the high-performance tumbler pigeons included infectious diseases and the sensory system; the main pathways significantly enriched in the large intestine of the high-performance tumbler pigeons mainly included cell growth and death as well as the digestive system; the pathways significantly enriched in the small intestine of the low-performance tumbler pigeons were generally less abundant, and those significantly enriched in the large intestine of the low-performance tumbler pigeons included membrane transport (membrane transport), etc. (Figure 4b). The annotation analysis results of the eggNOG database show that, apart from the unknown functions, amino acid transport and metabolism have the highest number, followed by carbohydrate transport and metabolism and transcription (Figure 5a). The extracellular structures, cell movement, and biosynthesis, transport and degradation of secondary metabolites were significantly enriched in the small intestine of the high-performance tumbler pigeon; the cell cycle control, cell division, chromosome distribution, replication, recombination and repair, as well as RNA processing and modification were significantly enriched in the ileum of the high-performance tumbler pigeon; the enriched functional items in the small intestine and ileum of the low-performance tumbler pigeon were generally lower (Figure 5b). The annotation analysis of the CAZy database revealed that the CAZy-dominant genes were assigned to 6 signaling pathways, including glycoside hydrolases, glycosyltransferases, carbohydrate-binding modules, carbohydrate esterases, auxiliary oxidoreductases, and polysaccharide lyases (Figure 6a). The intestinal segments of the high-performance tumbler pigeons were significantly enriched with active enzymes and polysaccharide hydrolases; the intestinal segments of the high-performance tumbler pigeons with low performance were significantly enriched with carbohydrate-binding modules, glycoside hydrolases and glycosyltransferases, etc.; the intestinal segments of the low-performance tumbler pigeons were significantly enriched with carbohydrate esterases, etc.; the carbohydrate enzymes in the intestinal segments of the low-performance tumbler pigeons were generally lower (Figure 6b). To identify biomarkers that show significant differences between groups, we conducted a LEfSe analysis to examine changes in the gut microbiota associated with high and low tumbler performance in tumbler pigeons after exercise (Figure 7). The results showed that there was 1 significantly different species in the cecal microbiota of high-performance tumbler pigeons (*p* < 0.05), which was *Sarcoscypha*; there were 6 significantly different species in the ileal microbiota of high-performance tumbler pigeons (*p* < 0.05), including *Bacillaceae*, *Bacillus*, *Pseudomonadota*, *Gizzard Lactobacillus*, and *Bacillus genus*, etc.; there were 9 significantly different species in the cecal microbiota of low-performance tumbler pigeons (*p* < 0.05), including *Cycloviridae*, *Circoviridae*, *Anaerobic fungi*, *Anaerobic fungus genus*, and *Fusarium genus*, etc.; there were 7 significantly different species in the ileal microbiota of low-performance tumbler pigeons, including *Acetococcus*, *Roundworm Virus family*, *Anaerobic fungi*, *Anaerobic fungus genus*, *Coccus-like bacteria*, *Mimic virus class*, and *Egertia class*, etc. We selected the bacterial genera and metabolites with significant differences in gut microbiota composition and in metabolite profiles, respectively, based on Spearman correlation coefficients, and plotted a heatmap (Figure 8). The results showed that in the positive mode of the jejunum, the genus *Corynebacterium* was significantly positively correlated with (4E)-2-oxoheptanoic acid, 2,3-dihydrofarnesol, 2-ethoxy-3-methylpyrazine, 6-hydroxypseudo-nicotinamide, etc., and significantly negatively correlated with fatty acylcarnitine and other metabolites (Figure 8a); in the negative mode of the jejunum, the metabolites of the *genus Corynebacterium* such as 2-deoxyinosine, 2-hydroxyheptanoic acid, and octadecanoic acid were significantly negatively correlated, while the metabolites of the *genus Salinispora* were significantly positively correlated with 1-(3-methylbutyryl)-6-vinylglucosamine (Figure 8b); in the positive mode of the ileum, the *genus Geobacter* was significantly positively correlated with glutamine-glycine and taraxacum diethylate (Figure 8c); in the negative mode of the ileum, the *genus Gigasphaera* was significantly positively correlated with 2-heptanone, hydroxychloroquine, and 1-phenyl-1-propanol (Figure 8d).

## 4. Discussion

### 4.1. Comparative Analysis of Blood Gas Indicators Before and After the High and Low Performance Tumbler Pigeons Exercises

Hb and Hct are important indicators that reflect the body’s cardiovascular gas and nutrient transport capacity and also evaluate the physiological state and metabolic regulation ability during exercise training [12]. Hct and Hb provide feedback based on the body’s nutritional status and the oxygen-carrying capacity of the blood. The higher the values within the normal range, the better the functional condition, which can enhance the body’s metabolic level during exercise, thereby improving athletic performance [10,13]. Hb also transports CO_2_, which can accelerate metabolic processes [14]. Ye et al. [15] conducted human trials. They found that individuals with high cardiac and pulmonary oxygen uptake had higher baseline Hb levels in the cerebral cortex, and that their neurocognitive responses improved after acute exercise, suggesting a correlation between the level of cardiac and pulmonary adaptation and local Hb content. The results of this study show that the concentrations of Hb and Hct in the serum of the tumbler pigeons in the HP group were significantly higher than those in the LP group. This difference may be related to the differences in the aerobic metabolic potential of the individuals in the two groups. The blood oxygen-carrying potential of the tumbler pigeons in the HP group was relatively higher, but the underlying regulatory mechanism still needs to be further verified. Studies have shown that higher Hb levels are significantly correlated with improved athletic performance. Hb, as an important iron-containing protein complex within red blood cells, plays a crucial role in the body’s gas transportation process, mainly responsible for the delivery of O_2_ and the transport of CO_2_ [16]. Higher Hb levels can provide tumbler pigeons with more O_2_ for transport and CO_2_ for removal within their bodies. This helps alleviate the fatigue caused by intense exercise in the pigeons.

Therefore, in this experiment, the Hb and Hct levels in the pigeons of the HP group were higher. The results of this study are largely consistent with those of previous studies. It is worth noting that the current research focuses on the changes in Hb in the local cerebral cortex. However, the intrinsic correlation between Hb and Hct in the serum after exercise and the animal’s movement performance still lacks a systematic analysis. PO_2_ is an indicator of gas exchange in the body’s tissues and lungs. It represents the driving force for gas exchange in the respiratory system. It can indicate the body’s respiratory acid–base balance and whether the organism is in an oxygen-deficient state. Wang et al. [17] found that after a 1000 m sprint, PCO2 levels in Yili horses’ blood were significantly lower than before the sprint. Michalik et al. [18] found, in a study of different performance-level motorcycle riders, that there was no significant difference in PCO2 levels in the blood of novice and advanced-level riders after the race. However, among advanced-level motorcycle riders, PCO2 levels in their blood after the race were negatively correlated with their season scores. In this study, the PO_2_ level in the serum of the tumbler pigeons in the HP group was significantly lower than that of the tumbler pigeons in the LP group. The reason might be that the tumbler pigeons in the HP group had stronger physical abilities and a faster muscle metabolic rate during movement, resulting in higher oxygen consumption and, subsequently, a lower PO_2_ level. However, the exact cause requires further investigation. The results of this study are largely consistent with those of previous studies. However, it should be noted that current research on blood gas indicators is mostly based on mammalian or human models. There are still few reports on its application in tumbler pigeons. Further in-depth exploration is still needed.

### 4.2. Comparative Analysis of Antioxidant Indexes Before and After High and Low Physical Activities of Tumbler Pigeons

Oxidative stress is a biological phenomenon in which the free radicals produced within the body are out of balance with the body’s antioxidant defense system [19]. Studies have shown that intense exercise increases free radical production, intensifying oxidative stress and causing cellular damage [20]. Antioxidants are substances that effectively inhibit oxidation reactions initiated by free radicals. They can directly and indirectly neutralize free radicals to counteract their oxidative damage to animal cells [21]. Exercise can induce the production of a large number of free radicals [22,23,24]. SOD and GSH-Px, etc., are important endogenous antioxidant enzymes in animals that play a significant role in eliminating free radicals and their metabolites [25]. CAT is an antioxidant enzyme in the body, mainly distributed in liver cells, red blood cells, and intestinal cells, accounting for approximately 40% of the total peroxisomal enzyme activity [26,27]. It can catalyze the decomposition of hydrogen peroxide, generating water and oxygen. Its main function is to eliminate oxygen free radicals in the body, inhibit lipid peroxidation, and reduce oxidative damage [28]. MDA is an important product of the oxidation and degradation of cell membrane lipids and a sensitive indicator of lipid peroxidation. Antioxidant indicators such as GSH-Px, SOD, and MDA can reflect the body’s oxidative status. When the body is affected by oxidative stress, the level of MDA increases. Lactate is the main product of anaerobic glycolysis during physical activity. During high-intensity, rapid movements, the rate of anaerobic glycolysis of glycogen increases, leading to a rise in Lac concentration in muscle cells and accumulation of Lac in the muscles [29]. The research has found that, after intense exercise, the mitochondrial respiratory chain of police dogs generates excessive reactive oxygen species, leading to the accumulation of MDA and an imbalance in antioxidant enzyme activity [30]. During intense exercise, the body’s aerobic respiration cannot meet energy demands, so it activates anaerobic glycolysis, leading to the accumulation of large amounts of lactic acid and an increase in the activity of related enzymes [31]. Tumbler pigeons involve short but intense physical exertion. The HP group had stronger tumbling ability. During high-intensity exercise, the pectoral muscles of these pigeons produced a large amount of Lac. This Lac was transported to the liver, where it was converted to glucose by lactate dehydrogenase for energy production. However, the catalytic reaction of lactate dehydrogenase would generate a large amount of reactive oxygen species.

To eliminate these reactive oxygen species, the liver would consume a large amount of SOD, GSH-Px, and CAT. The consumption rate was much higher than the liver’s immediate synthesis rate. The unremoved reactive oxygen species would attack the unsaturated fatty acids in the liver cell membranes, triggering lipid peroxidation. As a result, the MDA content in the bodies of the HP group pigeons was significantly higher than that of the LP group pigeons. At the same time, the antioxidant indicators were significantly lower than those of the LP group pigeons. It is worth noting that the research on antioxidant indicators after exercise has mainly focused on police dogs and athletes. There are currently few reports on this topic in pigeons. Further in-depth exploration is still needed.

### 4.3. Analysis of the Association Between Gut Metabolites and Microbiota in High and Low Physical Performance Tumbler Pigeons After Exercise

This experiment utilized metabolomics and metagenomics techniques to investigate the effects of high and low exercise performance on the intestinal microbiota and metabolic profiles of tumbler pigeons after exercise. It was found that tumbler pigeons significantly affected the biosynthesis of unsaturated fatty acids, starch and sucrose metabolism, and glycolysis. Moreover, in the HP group, the intestinal flora of tumbler pigeons, including *Corynebacteriaceae*, *Bacillus*, the *Pseudomonad phylum*, and *Corynebacterium*, showed significant differences as biomarkers. Trehalose is a non-reducing disaccharide composed of glucose molecules. It can protect the structure of biological molecules and maintain life processes [32]. The research has found that trehalose also possesses antioxidant properties, which can reduce the accumulation of hydrogen peroxide and oxygen free radicals in the body, and enhance the activities of CAT and SOD [33]. In this study, trehalose content in the ileum of tumbler pigeons in the HP group was significantly higher than in the LP group, suggesting a metabolic adaptation to the oxidative burden imposed by prolonged exercise. Intense exercise generates a large amount of reactive oxygen species, which can damage intestinal epithelial cells and disrupt the intestinal barrier function. Trehalose, as a cellular protective molecule, helps protect the intestinal epithelium from oxidative damage during and after exhaustive exercise by eliminating free radicals and stabilizing the cell membrane.

Furthermore, trehalose can act as a compatible solute, maintaining the osmotic pressure balance in the intestinal lumen under physiological stress. Therefore, the enrichment of trehalose indicates that the tumbler pigeons in the HP group have a stronger ability to buffer the intestinal oxidative stress induced by exercise, which may help maintain intestinal homeostasis and thereby support more stable flight performance. On the contrary, the key intermediate of the tricarboxylic acid (TCA) cycle, α-ketoglutarate, was significantly decreased in the flapping pigeons of the HP group. α-Ketoglutarate has multiple metabolic functions. It can produce key substrates for energy metabolism via the TCA cycle, serves as a nitrogen acceptor in amino acid transamination, and also provides a carbon skeleton for glutamine synthesis [34]. The depletion of α-ketoglutarate indicates an increase in its metabolic consumption rate. During high-intensity flight, the sudden increase in energy demand accelerates the flux of the TCA cycle, rapidly depleting the available α-ketoglutarate.

Furthermore, α-ketoglutaric acid can be converted to glutamate by transamination, and glutamine is synthesized by glutamine synthetase. During exhaustive exercise, the demand for glutamine may increase, as it is the main respiratory fuel for intestinal epithelial cells and plays a crucial role in maintaining intestinal barrier integrity and supporting epithelial repair after stress-induced injury [35,36]. Therefore, the depletion of α-ketoglutarate in the tumbler pigeons of the HP group may be partly due to its accelerated conversion to glutamine biosynthesis to meet the increased demand for intestinal epithelial maintenance and repair after intense exercise. The analysis of the correlation between metabolites and microorganisms revealed that octadecanoic acid showed a significant negative correlation with the *Corynebacterium genus*. Octadecanoic acid, as a fatty acid, participates in the lipid metabolism process within the body. It undergoes β-oxidation to generate acetyl coenzyme A and enters the tricarboxylic acid cycle to provide energy for muscle contraction [37]. In the HP group, the tumbler pigeons required a higher energy supply during high-intensity exercise. After being metabolized through β-oxidation, octadecanoic acid is converted into acetyl coenzyme A and enters the tricarboxylic acid cycle. As energy metabolism occurs, octadecanoic acid is consumed in large quantities. Therefore, the content of octadecanoic acid in the jejunum of the HP group pigeons was significantly lower than that of the LP group. The *genera Bacillus* and *Pseudomonas*, among others, can degrade polysaccharides [38]. Both genera can produce cellulase, which breaks down cellulose in the substrate into monosaccharides [39,40]. Therefore, they have a strong ability to degrade cellulose. In this experiment, the *genus Bacillus* and the order *Pseudomonadales* were significantly enriched in the intestines of the pigeons in the HP group. It is speculated that the pigeons in the HP group can utilize the enriched bacterial flora in their intestines to synthesize and secrete cellulase in large quantities, efficiently decompose the indigestible cellulose in the feed, increase the utilization rate of crude fiber, enhance the utilization of carbohydrates by the body, and improve the energy supply level.

## 5. Conclusions

Under these experimental conditions, there were significant differences in blood gas indicators, antioxidant performance, intestinal metabolites, and microbial flora among tumbler pigeons with different exercise performance groups. At the same time, exercise activated various metabolic pathways in the tumbler pigeons, mainly through pathways such as biosynthesis of unsaturated fatty acids, starch and sucrose metabolism, and glycolysis. The *Bacillus genus* and *the Pseudomonad phylum*, among others, are biomarkers that show significant differences in the intestines of tumbler pigeons in the HP group. The intestinal microbial flora of the tumbler pigeon in the HP group exhibited greater advantages in carbohydrate metabolism and other functions, demonstrating a stronger ability to degrade polysaccharides. However, the underlying biological mechanisms still need to be confirmed through targeted mechanism studies.

## Figures and Tables

**Table 1:** Differences in blood gas indicators between high and low performance tumbler pigeons.

Index	HP	LP	*p*-Value
pH	7.61 ± 0.027	7.57 ± 0.024	0.311
PvCO_2_/(mmHg)	23.22 ± 0.807	22.67 ± 1.143	0.708
PO_2_/(mmHg)	44.10 ± 1.048 ^a^	55.00 ± 3.614 ^b^	0.015
Na^+^/(mmol/L)	144.70 ± 1.169	141.42 ± 1.249	0.073
K^+^/(mmol/L)	4.28 ± 0.116	4.06 ± 0.185	0.354
Cl^−^/(mmol/L)	129.60 ± 2.673	123.49 ± 3.355	0.182
Ca^2+^/(mmol/L)	1.12 ± 0.012	1.14 ± 0.021	0.387
Hct/%	42.60 ± 0.933 ^A^	37.67 ± 0.995 ^B^	0.002
TCO_2_/(mmol/L)	24.04 ± 1.282	21.33 ± 0.899	0.092
Hb/(g/dL)	14.43 ± 0.326 ^A^	12.84 ± 0.328 ^B^	0.003
HCO_3_^−^/(mmol/L)	23.38 ± 1.281	20.66 ± 0.890	0.089
BE/(mmol/L)	4.64 ± 0.749	3.17 ± 0.545	0.120
SO_2_/%	88.18 ± 0.948	91.01 ± 1.743	0.194

Notes: HP = high flight performance tumbler pigeons (*n* = 10); LP = low flight performance tumbler pigeons (*n* = 12). After formal blood collection, two pigeons in the HP group were excluded from subsequent analysis: one sample exhibited severe hemolysis, and another showed abnormal outlier values confirmed by Grubbs’ outlier test. Finally, the effective sample size was *n* = 10 for the HP group and *n* = 12 for the LP group. pH, potential of hydrogen; PvCO_2_, partial pressure of venous carbon dioxide; PO_2_, partial pressure of oxygen; Na^+^, sodium ion; K^+^, potassium ion; Cl^−^, chloride ion; Ca^2+^, calcium ion; Hct, hematocrit; TCO_2_, total carbon dioxide; Hb, hemoglobin; HCO_3_^−^, bicarbonate; BE, base excess; SO_2_, oxygen saturation. Data are presented as mean ± standard error of the mean (SEM). Values within the same row with no superscript letters or identical superscripts indicate no significant difference (*p* > 0.05). Values with different lowercase letters indicate significant differences (a, b: *p* < 0.05), and those with different uppercase letters indicate highly significant differences (A, B: *p* < 0.01).

**Table 2:** Differences in Liver Antioxidant Indicators Before and After Flight in High- and Low-Performance Tumber Pigeons.

Index	HP	LP	*p*-Value
T-AOC/(μmol/g)	0.47 ± 0.047	0.55 ± 0.044	0.253
GSH-Px/(ng/mL)	8.57 ± 0.265 ^A^	15.27 ± 0.238 ^B^	<0.001
SOD/(ng/mL)	9.4 ± 0.393 ^A^	17.56 ± 0.339 ^B^	<0.001
MDA/(nmol/mL)	6.99 ± 0.097 ^A^	4.16 ± 0.091 ^B^	<0.001
Lac/(μmol/g)	0.05 ± 0.003 ^a^	0.04 ± 0.002 ^b^	0.019
TP/(g/L)	3.81 ± 0.174	3.97 ± 0.172	0.515
CAT/(pg/mL)	407.34 ± 13.933 ^A^	750.63 ± 16.319 ^B^	<0.001

Notes: HP = high flight performance tumbler pigeons (*n* = 9); LP = low flight performance tumbler pigeons (*n* = 10). T-AOC, total antioxidant capacity; GSH-Px, glutathione peroxidase; SOD, superoxide dismutase; MDA, malondialdehyde; Lac, lactic acid; TP, total protein; CAT, catalase. Data are presented as mean ± standard error of the mean (SEM). Values within the same row with no superscript letters or identical superscripts indicate no significant difference (*p* > 0.05). Values with different lowercase letters indicate significant differences (a, b: *p* < 0.05), and those with different uppercase letters indicate highly significant differences (A, B: *p* < 0.01).

**Table 3:** Alpha diversity of the jejunum and ileum of tumbler pigeons with high and low exercise performance.

Index	LFK	HFK	*p*-Value	LFI	HFI	*p*-Value
Chao1	666.78 ± 33.194 ^a^	782.75 ± 31.182 ^b^	0.012	706.11 ± 16.152	777.28 ± 39.147	0.114
Shannon	2.95 ± 0.009	2.97 ± 0.009	0.398	2.99 ± 0.023	3.00 ± 0.013	0.456
Simpson	0.88 ± 0.002	0.88 ± 0.002	0.702	0.89 ± 0.003	0.89 ± 0.003	0.877

Notes: HFK = HB group tumbler pigeon jejunum; LFK = LB group tumbler pigeon jejunum; HFI = HB group tumbler pigeon ileum; LFI = LB group tumbler pigeon ileum. Values are presented as mean ± standard error of the mean (SEM). Different lowercase superscript letters (a, b) within the same row indicate significant differences between groups at *p* < 0.05; identical superscript letters denote no significant difference. The *p*-value represents the significance level of the intergroup comparison for each alpha diversity index. Chao1, Shannon, and Simpson are common indicators of microbial alpha diversity.

## Data Availability

The data supporting the findings of this study are available from the corresponding author upon reasonable request.

## Supplementary Materials

The following supporting information can be downloaded at: https://www.mdpi.com/article/10.3390/biology15141193/s1, Figure S1: Total PCA score plot in positive and negative ion modes; HFK: HP group tumbler pigeon jejunum; HFI: HP group tumbler pigeon ileum; LFK: LP group tumbler pigeon jejunum; LFI: LP group tumbler pigeon ileum, The legends for all panels in the supplementary figures are identical; QC: quality control samples; (a) PCA score plot of metabolites in positive ion mode; (b) PCA score plot of metabolites in negative ion mode; Figure S2: OPLS-DA score plots and permutation test results of metabolite profiles under positive and negative ion modes; (a–d) Positive ion mode; (e–h) Negative ion mode; (a,e) OPLS-DA score plot between HFI and LFI groups; (b,f) Corresponding permutation test; (c,g) OPLS-DA score plot between HFK and LFK groups; (d,h) Corresponding permutation test; Figure S3: Clustering heatmaps of differential metabolites in positive and negative ion modes; (a,b) Positive ion mode; (c,d) Negative ion mode; (a,c) Differential metabolites between HFK and LFK groups; (b,d) Differential metabolites between HFI and LFI groups; Figure S4: PCA score plot of intestinal microbiota; Figure S5: ANOSIM analysis results of intestinal microbiota in tumbler pigeons; (a) ANOSIM test for jejunal microbiota between HFK and LFK groups; (b) ANOSIM test for ileal microbiota between HFI and LFI groups; Table S1: Selected differentially expressed metabolites; Table S2: Data Quality Control; Table S3: Total metabolites.
