# Supplementary material for: Comparative Study on Blood Gas Indicators, Antioxidant Capacity, Intestinal Metabolome, and Microbiome in High- and Low-Performance Tumbler Pigeons
Source: Biology (Basel). 2026 Jul 20;15(14):1193. doi: 10.3390/biology15141193 (PMC13405826; doi:10.3390/biology15141193)
Supplement: Supplementary file 1 [file biology-15-01193-s001.zip › Supplementary Figures.pdf]

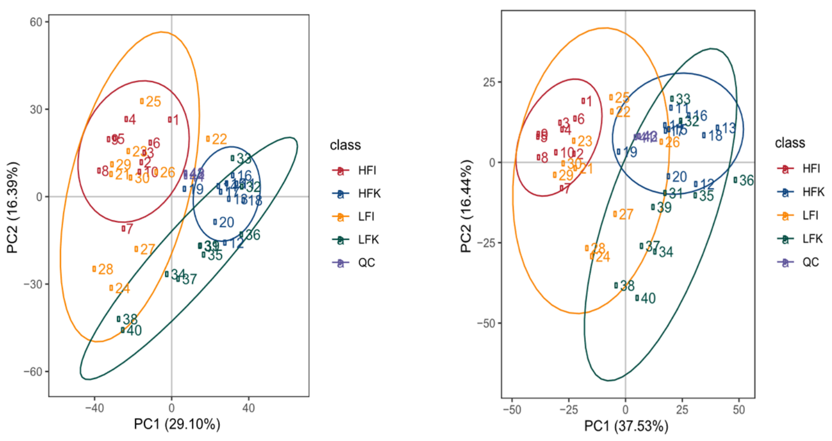

(a) (b)

Figure S1. Total PCA score plot in positive and negative ion modes; HFK: HP group tumbler pigeon jejunum; HFI: HP group tumbler pigeon ileum; LFK: LP group tumbler pigeon jejunum; LFI: LP group tumbler pigeon ileum, The legends for all panels in the supplementary figures are identical; QC: quality control samples; (a) PCA score plot of metabolites in positive ion mode; (b) PCA score plot of metabolites in negative ion mode.

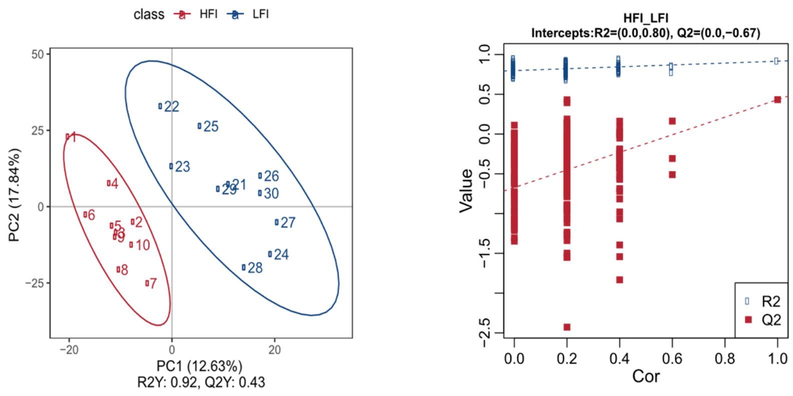

(a) (b)

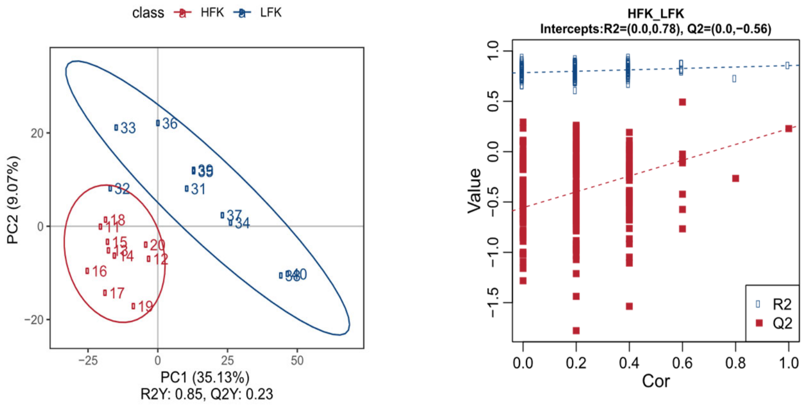

(c) (d)

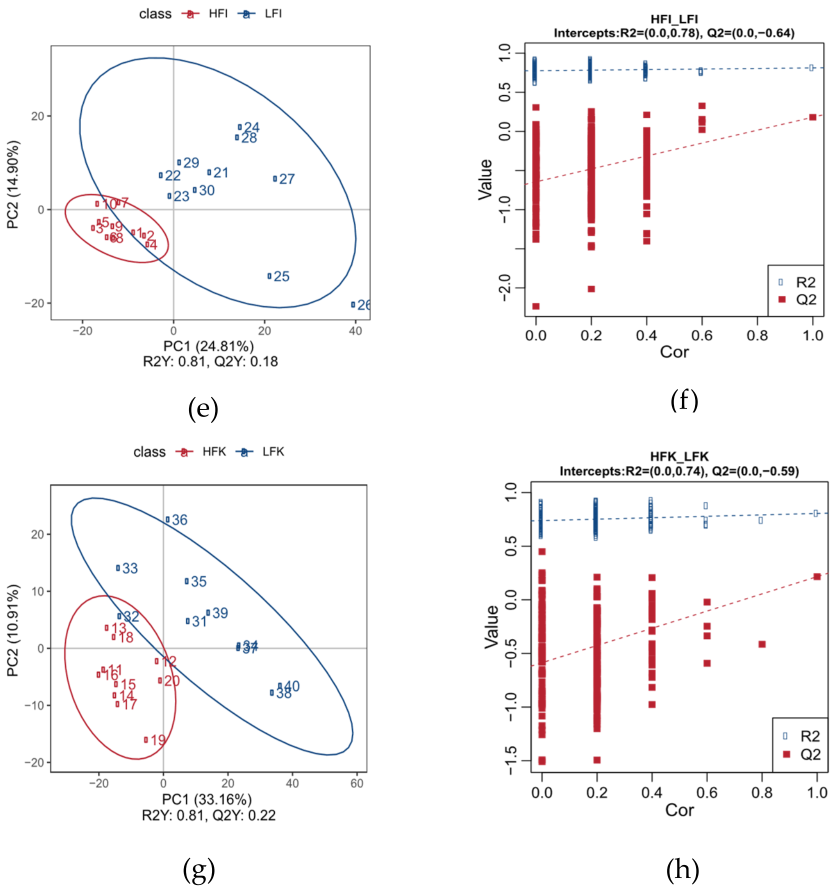

Figure S2. OPLS-DA score plots and permutation test results of metabolite profiles under positive and negative ion modes; (a–d) Positive ion mode; (e–h) Negative ion mode; (a,e) OPLS-DA score plot between HFI and LFI groups; (b,f) Corresponding permutation test; (c,g) OPLS-DA score plot between HFK and LFK groups; (d,h) Corresponding permutation test.

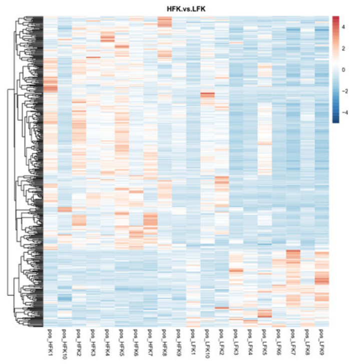

(a)

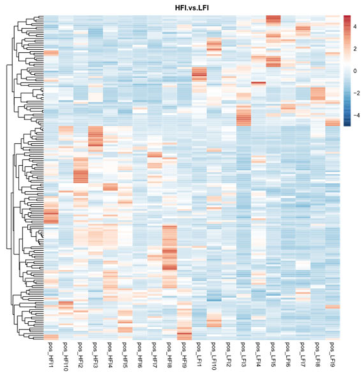

(b)

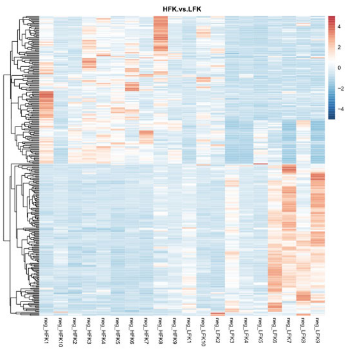

(c)

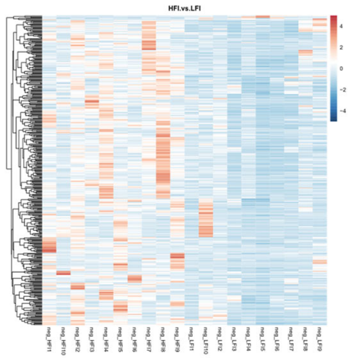

(d)

Figure S3. Clustering heatmaps of differential metabolites in positive and negative ion modes; (a,b) Positive ion mode; (c,d) Negative ion mode; (a,c) Differential metabolites between HFK and LFK groups; (b,d) Differential metabolites between HFI and LFI groups.

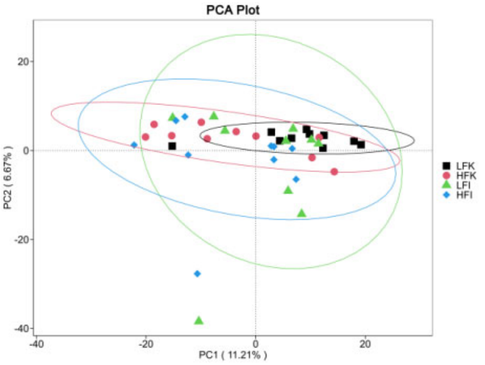

Figure S4. PCA score plot of intestinal microbiota.

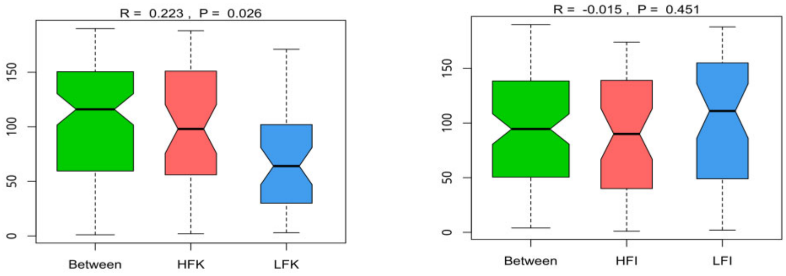

(a) (b)

Figure S5. ANOSIM analysis results of intestinal microbiota in tumbler pigeons; (a) ANOSIM test for jejunal microbiota between HFK and LFK groups; (b) ANOSIM test for ileal microbiota between HFI and LFI groups.
